# Novel tools and strategies for breaking schistosomiasis transmission: study protocol for an intervention study

**DOI:** 10.1186/s12879-021-06620-8

**Published:** 2021-09-30

**Authors:** Lydia Trippler, Jan Hattendorf, Said Mohammed Ali, Shaali Makame Ame, Saleh Juma, Fatma Kabole, Stefanie Knopp

**Affiliations:** 1 Swiss Tropical and Public Health Institute, Socinstrasse 57, 4051 Basel, Switzerland; 2 University of Basel, Petersplatz 1, 4001 Basel, Switzerland; 3 Public Health Laboratory - Ivo de Carneri, Wawi, P.O. Box 122, Chake-Chake, Pemba United Republic of Tanzania; 4 Neglected Diseases Program, Ministry of Health, P.O. Box 236, Zanzibar, United Republic of Tanzania

**Keywords:** Adaptive interventions, Behaviour change, *Bulinus*, Case finding, Diagnostics, Elimination, Hotspot, Interruption of transmission, Schistosomiasis, *Schistosoma haematobium*, Snail control, Surveillance-response, Zanzibar

## Abstract

**Background:**

Global elimination of schistosomiasis as a public health problem is set as target in the new World Health Organization’s Neglected Tropical Diseases Roadmap for 2030. Due to a long history of interventions, the Zanzibar islands of Tanzania have reached this goal since 2017. However, challenges occur on the last mile towards interruption of transmission. Our study will investigate new tools and strategies for breaking schistosomiasis transmission.

**Methods:**

The study is designed as an intervention study, documented through repeated cross-sectional surveys (2020–2024). The primary endpoint will be the sensitivity of a surveillance-response approach to detect and react to outbreaks of urogenital schistosomiasis over three years of implementation. The surveys and multi-disciplinary interventions will be implemented in 20 communities in the north of Pemba island. In low-prevalence areas, surveillance-response will consist of active, passive and reactive case detection, treatment of positive individuals, and focal snail control. In hotspot areas, mass drug administration, snail control and behaviour change interventions will be implemented. Parasitological cross-sectional surveys in 20 communities and their main primary schools will serve to adapt the intervention approach annually and to monitor the performance of the surveillance-response approach and impact of interventions. *Schistosoma haematobium* infections will be diagnosed using reagent strips and urine filtration microscopy, and by exploring novel point-of-care diagnostic tests.

**Discussion:**

Our study will shed light on the field applicability and performance of novel adaptive intervention strategies, and standard and new diagnostic tools for schistosomiasis elimination. The evidence and experiences generated by micro-mapping of *S. haematobium* infections at community level, micro-targeting of new adaptive intervention approaches, and application of novel diagnostic tools can guide future strategic plans for schistosomiasis elimination in Zanzibar and inform other countries aiming for interruption of transmission.

*Trial registration* ISRCTN, ISCRCTN91431493. Registered 11 February 2020, https://www.isrctn.com/ISRCTN91431493

## Background

Schistosomiasis is a neglected tropical disease (NTD) with a considerable impact on global health [1, 2]. Since the mid-1980s, efforts in endemic countries mainly focused on the control of morbidity using preventive chemotherapy with praziquantel [3]. A paradigm shift occurred in recent years: in 2012, the World Health Organization (WHO) declared as goal to eliminate schistosomiasis as a public health problem and to interrupt transmission in selected areas by 2025 [4]. In the new WHO Roadmap on NTDs published in 2021, the global elimination of schistosomiasis as a public health problem and the validated absence of infections in humans in 25 among 78 endemic countries are set as targets for 2030 [5].

**Fig. 1:**
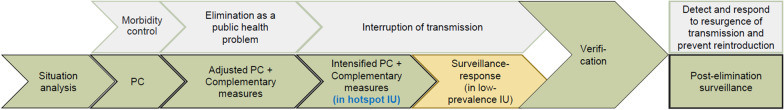
Programmatic steps to control and eliminate schistosomiasis. In green and gray text boxes: recommendations according to WHO [4]; in blue fonts and yellow text box: additional components of the SchistoBreak study. PC: preventive chemotherapy; IU: implementation unit

There are several countries and areas that have made great progress in the fight against schistosomiasis in the past and may aim to achieve full interruption of transmission in the next few years [6–10]. These countries will need clear guidance on which intervention strategies to apply, which population groups to target, which diagnostics to use, and at what thresholds to change and adapt their strategies [11–13]. Moving from morbidity control towards elimination as public health problem and interruption of transmission, WHO recommends in their schistosomiasis progress report 2001–2011 and strategic plan 2012–2020, the intensification of mass drug administration (MDA) and the implementation of complementary public-health interventions in addition to preventive chemotherapy (Fig. 1) [4]. In World Health Assembly resolution 65.21 (05/12) these complementary interventions are indicated as “strengthened health systems, […] provision of water and sanitation, as well as hygiene education and snail control” [14]. In the new WHO NTD Roadmap 2021–2030, besides MDA, the following core strategic interventions against schistosomiasis are listed: Water, Sanitation and Hygiene (WASH), vector control, veterinary public health, case management and other interventions such as behaviour change, self-care and environmental management [5]. Finally, according to WHO, once interruption of transmission is close or has been achieved, affected countries will need to implement a surveillance system “to detect and respond to resurgence of transmission and to prevent reintroduction from regions where the disease is still endemic” [4]. Despite these recommendations, specific guidance and thresholds on when, where and how to adapt intervention strategies in near-to-elimination settings is yet to be developed [11, 12, 15]. More evidence on the feasibility, impact, effectiveness and sustainability of multi-pronged intervention approaches needs to be generated.

A wealth of experience and insights regarding multi-disciplinary interventions and research for urogenital schistosomiasis elimination was gained over the past decade from 2011 to 2020 within the Zanzibar Elimination of Schistosomiasis Transmission (ZEST) project [6, 16–19]. The islands Pemba and Unguja, belonging to the Zanzibar archipelago of the United Republic of Tanzania, used to be highly endemic for urogenital schistosomiasis in the past century [20–22]. To combat urogenital schistosomiasis, caused by *Schistosoma haematobium*, on the islands, regular MDA campaigns with praziquantel administration to schoolchildren and the whole community were implemented since the 1980s [23–26]. In 2010, Zanzibar had managed to reach low prevalences of *S. haematobium* infections and subsequently committed to eliminate urogenital schistosomiasis from the islands [17, 18]. From 2012 till 2017, in addition to biannual MDA, some randomised areas received interventions with chemical mollusciciding to target *Bulinus globosus,* the intermediate host snail for *S. haematobium* in Zanzibar [27, 28] and behaviour change measures to reduce people`s freshwater contact [17, 19]. In 2017, most areas on the Zanzibar islands had eliminated urogenital schistosomiasis as a public health problem (defined as < 1% heavy intensity infections) [5, 17, 18]. However, transmission of *S. haematobium* infections was not yet interrupted and several challenges were identified on the way towards schistosomiasis elimination.

These challenges included a marked temporal and spatial heterogeneity of *S. haematobium* prevalence across the islands, as well as the application of standard diagnostic tests with insufficient sensitivity to detect light intensity infections [6, 17, 18, 29–32]. While most of the study sites showed continuously low prevalences from 2011/12 to 2020, some “hotspot” areas with consistent or recurring moderate or high prevalences existed [6, 17, 18]. To address this heterogeneity and to sustain and accelerate the gains made towards elimination, there is a need to target and regularly adapt interventions to the local micro-epidemiology [12, 17, 18].

In low-prevalence areas, it will be important to progress towards complete interruption of transmission by applying an intervention approach that enables the reliable identification and treatment of all cases and thus prevents outbreaks and recrudescence, without overtreating a mostly healthy population [18] and thereby risking treatment fatigue and/or the development of resistance against praziquantel [33]. The participation of countries in the development and use of surveillance-response approaches to ensure that the progress made is sustained and advanced has been stressed in several publications [15, 34–39].

In hotspot areas that are often characterized by a large number of water bodies containing the intermediate host snails and proximity of households and schools to transmission sites [40, 41] there is a need to tackle the persistent transmission to progress towards elimination using a comprehensive package of interventions, including MDA, but also other interventions such as snail control and behaviour change communication and WASH measures [11, 17, 18, 32].

Accurate, reliable and affordable diagnostic tools are an essential requirement for NTD programmes and have been identified as a priority area for critical action in the WHO NTD Roadmap 2021–2030 [5, 13, 15, 42, 43]. Moving towards schistosomiasis elimination, there is an enhanced need for sensitive and specific diagnostic tests that are high-throughput, and affordable and applicable at the point of care to assess reliably *S. haematobium* infections, prevalences and incidence [13, 43]. An accurate picture of the endemic situation is important for programmatic decision making, to tailor specific intervention packages in line with (yet to be developed) target thresholds to those in need, to determine correctly the performance and impact of interventions in elimination settings, and to document sustained elimination [13, 29–31, 44].

In our protocol, we describe an implementation research study that will address the focality and heterogeneity of *S. haematobium* transmission, using novel adaptive intervention strategies as well as standard and new diagnostic tools for schistosomiasis elimination in Zanzibar.

## Methods/design

### Study aim

The overall objective of the study is to investigate new tools and strategies for breaking schistosomiasis transmission.

### Primary and secondary objectives

The primary objective of this study is to quantify the sensitivity of an adaptive surveillance-response approach for its ability to detect *S. haematobium* infected individuals in low-prevalence areas to trigger an appropriate intervention response. The primary outcome variable will be the number of *S. haematobium* infected individuals detected and reported through the surveillance approach divided by the number of positive individuals in the population as extrapolated from the cross-sectional surveys, i.e. the mean sensitivity of the surveillance-response approach determined over 3 years.

Secondary outcome analyses will cover additional performance parameters of the surveillance approach, as defined by WHO [45]. Moreover, we will assess the impact of multi-disciplinary interventions in hotspot areas, as well as the coverage of test-and-treat activities in low-prevalence areas and of MDA in hotspot areas. Other secondary outcomes will include the accuracy of several diagnostic approaches and micro-mapping of characteristics of the study shehias, including the number and location of schools, madrassas (Islamic schools), and health facilities, the number and location of water bodies and the abundance of intermediate host snails of the genus *Bulinus*.

### Study design

The study is designed as intervention study, documented through repeated cross-sectional surveys to assess the performance of the surveillance-response approach.

### Study setting

**Fig. 2:**
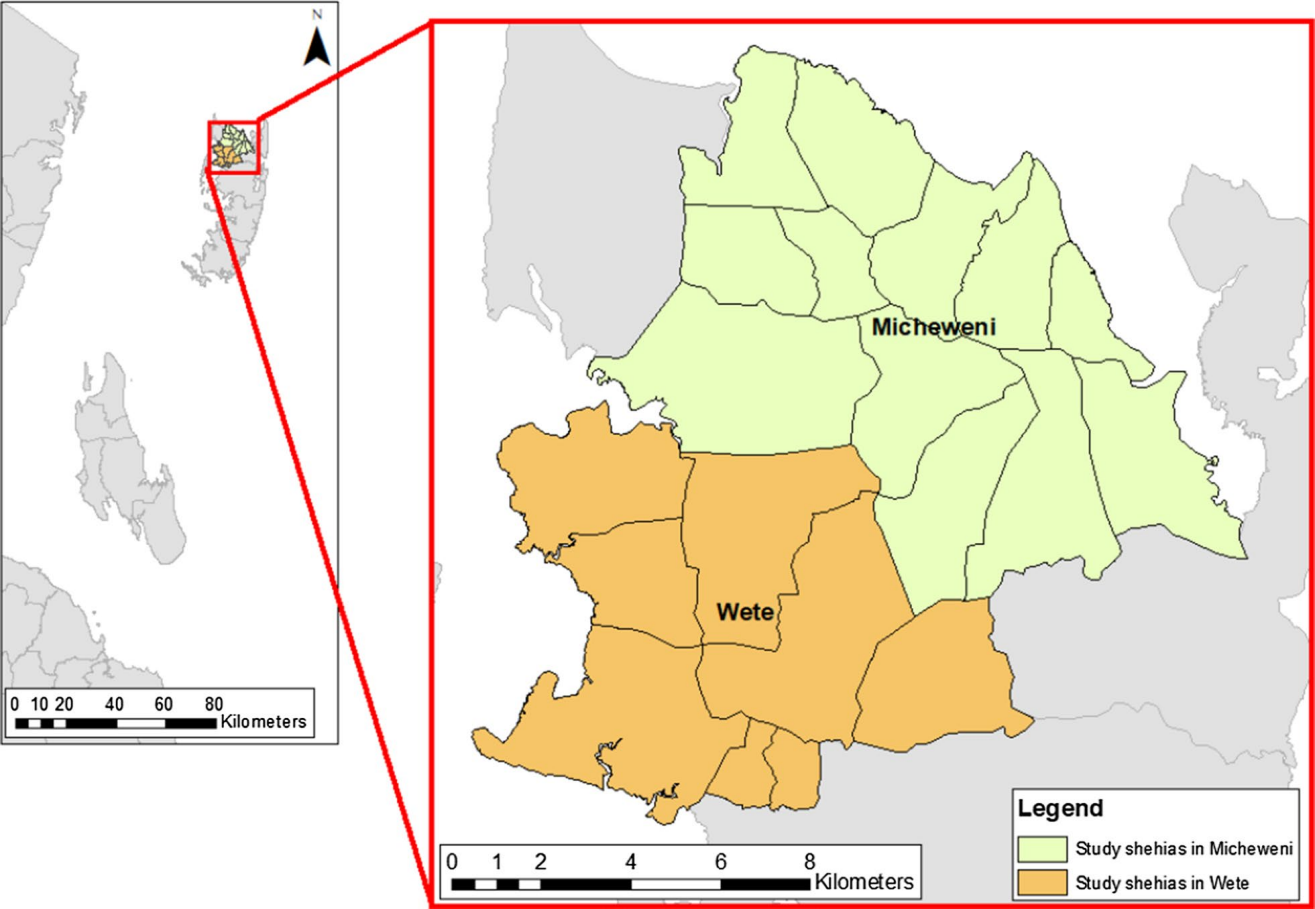
Twenty study shehias (implementation unit) of the SchistoBreak project in Wete and Micheweni districts in Pemba, Tanzania. The image base map (United Republic of Tanzania – Subnational administrative boundaries) was downloaded from OCHA services (https://data.humdata.org/dataset/tanzania-administrative-boundaries-level-1-to-3-regions-districts-and-wards-with-2012-population). The data source is: Tanzania National Bureau of Statistics/UN OCHA ROSA. The data are published under the following license: Creative Commons Attribution for Intergovernmental Organisations (CC BY-IGO; (https://creativecommons.org/licenses/by/3.0/igo/legalcode)). Additionally, we received written permission to use and adapt the data from the UN Office for the Coordination of Humanitarian Affairs (OCHA). Additional shape files for the map (shehia boundaries) were provided by the Zanzibar Commission of Lands to the Zanzibar Neglected Diseases Programme

The implementation research study with the acronym “SchistoBreak” is a joint project with partners from the Swiss Tropical and Public Health Institute (Swiss TPH), Basel, Switzerland the Public Health Laboratory-Ivo de Carneri (PHL-IdC), Pemba, United Republic of Tanzania and the Neglected Diseases Programme of the Ministry of Health, Social Welfare, Elderly, Gender, and Children (MoHSWEGC) Zanzibar, United Republic of Tanzania. The fieldwork for the SchistoBreak study will be conducted in Pemba, an island that forms part of the Zanzibar archipelago, from March 2020 to June 2024. The population density of Pemba was 530 people/km^2^ in 2012 and the projected population for 2019 was around 500,000 people [46]. The study area will consist of 20 shehias (small administrative areas) located in the rural districts Micheweni and Wete in the north of Pemba (Fig. 2). The average population size in the study shehias is ~ 3,900 individuals per shehia [47]. To address the small-scale heterogeneity in the study area and to micro-target implementation, not the districts but each shehia will serve as one implementation unit (IU). The 20 study shehias were selected based on their isolated but contiguous location in the north of Pemba and very good documentation of *S. haematobium* prevalences in 10 among the 20 shehias from 2012 to 2020 [6, 17, 18].

The study will take place in communities, schools and health facilities. In Zanzibar, public primary schools contain the grades 1–6 and some schools include a nursery school. In addition, most of the children in Zanzibar visit a madrassa [48]. The net enrolment rate in primary schools was 68.4% in Micheweni and 81.9% in Wete in 2014/15 [49].

### Participant eligibility

All individuals who meet the following inclusion criteria are eligible to participate in the study:

1. All persons aged ≥ 4 years, living in the study shehias.
2. Submitted informed consent form (ICF) signed by parent or legal guardian in case of participating children and adolescents, or signed by the participant in case of participating adults, and submitted assent form in case of children aged 12 years and older signed by the participating children.
3. Urine sample with sufficient volume to perform diagnostic tests provided.

All individuals who do not meet the following exclusion criteria are not eligible to participate in the study:

1. Children < 4 years.
2. Children, adolescents and adults not living in the study area.
3. ICF not submitted or not signed by parent or legal guardian in case of participating children or adolescents or not signed by the participant in case of participating adults. Assent form not submitted or not signed by the child in case of participating children aged 12 years or older.
4. No urine sample of sufficient volume to perform diagnostic tests provided.

### Shehia characteristics survey

Upon start of the SchistoBreak project, we will conduct a survey to gain an aggregate picture of the characteristics of the 20 study shehias. In a first step, in each shehia, we will meet with the sheha (head of shehia) and invite him to participate in a questionnaire interview to collect data about the population size, the number and type of schools, natural open freshwater bodies, health facilities and public clean water sources available in each shehia. Subsequently, with the sheha’s permission and the support of an assistant sheha, we will visit all nursery, primary and secondary schools and all madrassas in the shehia and assess the geolocation, type of school, and the number of children enrolled in the school. Moreover, we will visit all known human water contact sites (HWCSs) at the natural open freshwater bodies in the shehias and collect data of locality, type and characteristics of the water body, regular behavioural activities at the water bodies and the abundance of freshwater snail species and specifically the intermediate host snail *Bulinus*. Finally, we will visit all health facilities in the shehias and determine the location and type of facility, i. e. whether they are of public or private status and offer mother and child health care services. For all processes of data collection during the shehia characteristics survey, we will use Open Data Kit (ODK) software (www.opendatakit.org), installed on a computer tablet (Samsung Galaxy Tab A 2019).

### Annual cross-sectional parasitological surveys

The annual cross-sectional surveys will be implemented in all 20 communities and their main public primary schools at sub-district shehia level. This micro-mapping approach will allow us to determine *S. haematobium* prevalences in the school-aged population, which is at highest risk of schistosomiasis, as well as in the whole community to get an accurate picture of infection levels in preschool-aged children, school-aged children, adolescents and adults. The cross-sectional surveys will serve to stratify the study area into low-prevalence and hotspot IUs and hence to micro-target the interventions according to pre-set prevalence thresholds. Moreover, the cross-sectional surveys will allow to determine and monitor the performance of the surveillance-response approach and impact of interventions over the study period. Participants of the annual cross-sectional parasitological surveys identified as infected with *S. haematobium* will be offered treatment with praziquantel (40 mg/kg using a dose pole) [50].

### Annual community-based parasitological surveys

#### Selection of houses and participants

For the annual community-based surveys, 70 housing structures per shehia will be selected by a computer-based randomisation procedure from Geographical Information System (GIS) shape files provided by the Zanzibar Mapping Initiative (http://www.zanzibarmapping.com) of the Commission of Lands Zanzibar. Randomly selected housing structures will be located by field enumerators by using the navigation app Maps.me (https://www.maps.me/) combined with ODK installed on a computer tablet (Samsung Galaxy Tab A 2019). Accounting for a 30% dropout of housing structures that may not be inhabited (i.e. shops, sheds, mosques, houses under construction or abandoned houses) and households without any member willing to participate, we estimate that we will have a final sample size of 50 houses per shehia included in the survey. People sharing the same kitchen or pot will define a household. All household members meeting the inclusion criteria will be invited to participate in the survey and to provide one own urine sample, which will be examined for *S. haematobium* infection markers. With an average of five people per household, we estimate to collect urine samples from a total of 250 participants per shehia. Additionally, one adult household member present at the first visit to the house will be invited to participate in a questionnaire survey to assess household characteristics. If more than one adult is present and eligible to participate at first visit, we will randomly select the participant of the questionnaire survey by using a playing-cards approach.

#### Data collection

A field enumerator using ODK and the wayfinding app Maps.me will visit each selected housing structure. The geolocation of each housing structure will be recorded in ODK. If the house is inhabited, the study will be explained in lay terms to the present household members and they will be invited to participate in the survey. Information and consent forms (ICF) for all eligible household members and assent forms for children aged 12 to 18 years will be distributed. Once adult participants and in the case of children aged below 18 years their parents provided written informed consent by signing the form and, additionally, once children aged 12 to 18 years agreed to participate by signing the child assent form, plastic containers (100 ml) for urine collection will be distributed for each participant. The plastic containers will be labelled with a unique identifier code and an individual sticker picture, which both match the same identifier code and sticker picture on a provided paper form containing the household member names to support their correct identification. Additionally, one adult household member will be invited to answer several questions related to participation in the last MDA, water contact behaviour, access to safe water sources, opinion regarding intervention approaches against schistosomiasis, and the number of individuals living in the household and their main demographic information, such as sex and age. On the following day, the study team will revisit the households and collect the (remaining) signed ICFs, signed assent forms and filled urine containers. Each shehia will be visited for three days, to cover all selected houses and ensure maximal participation and compliance with urine collection.

### School-based parasitological surveys

#### Selection of participants

The annual school-based cross sectional survey will be conducted in the main public primary school of each shehia. If a shehia has several public primary schools, the school with most students will be surveyed. If a shehia has no public primary school, no school will be surveyed. In each of the schools, a total of 175 students aged 4–17 years will be randomly selected for urine collection. For this purpose, one class of each nursery and standard 1–6 grades, will be selected based on computer-randomised lists. In the selected classes, all children will line up, stratified by sex. Subsequently, we will systematically select each third child in the lines to be included in the study until 25 children per class are reached. This procedure will be continued until we reach a total of 175 selected children from the 7 selected grades. Accounting for a 20% drop-out due to non-consenting parents, absenteeism of children or inability to produce a urine sample of sufficient volume, we aim for a final sample size of 20 children per standard (i.e. a total of 140 students per school).

#### Data collection

Each selected child will be provided with an ICF for their parents to read and sign. Each selected child aged 12 years or older will additionally be invited to sign an assent form on its own behalf. On the following day, once signed ICFs and assent forms are submitted, children will be registered, including information about their age, sex, travel history and participation in the last round of MDA. Subsequently, children will be handed over a plastic container labelled with a unique identifier code and invited to produce their own urine sample, which will be collected by the study team on the same morning. In addition, participants from grade 3, 4, and 5 will be invited to participate in a questionnaire interview about the knowledge, attitudes and practices regarding schistosomiasis transmission and prevention (KAP). The children will be asked about their knowledge of the animals that are part of the *S. haematobium* lifecycle, their own water contact behaviour, access to safe water sources, and their knowledge about opportunities to prevent both getting and spreading schistosomiasis.

### Interventions and data collection

**Fig. 3:**
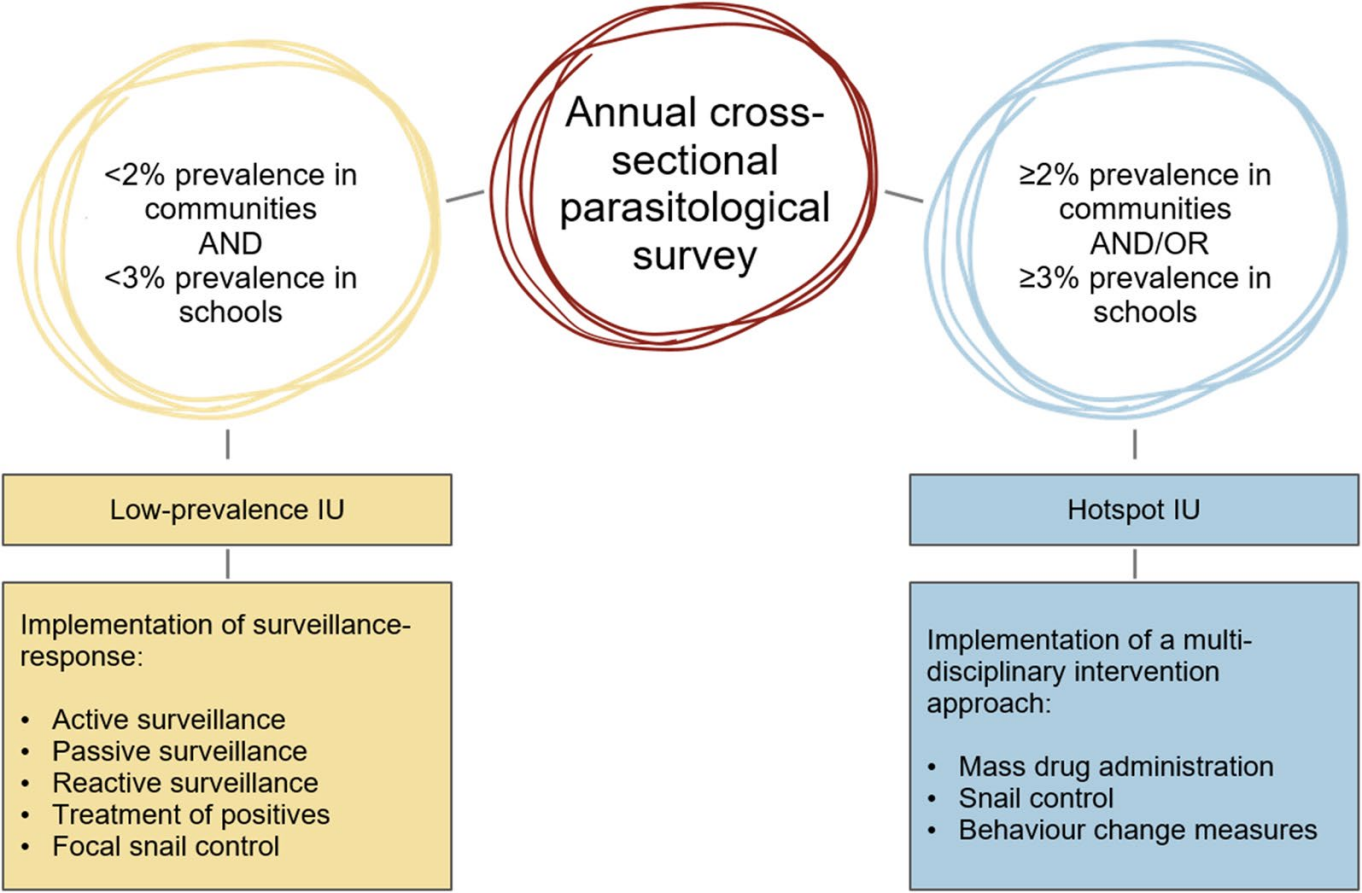
Classification of the SchistoBreak study shehias in Pemba, Tanzania, into low-prevalence and hotspot implementation units (IUs) based on adaptive *S. haematobium* prevalence thresholds

In the study, specific intervention packages will be tailored and adapted to the local micro-epidemiology of urogenital schistosomiasis in the shehias. Low-prevalence IUs will receive tailored surveillance-response measures, consisting of active, passive and reactive case finding, treatment of *S. haematobium*-positive individuals and focal snail control. Hotspot IUs will receive a multi-disciplinary intervention package including MDA, snail control and behaviour change communication. The classification of the study shehias into low-prevalence or hotspot IUs will be based on the results of annual cross-section surveys. Initially, a shehia will be considered a low-prevalence IU if it shows an apparent *S. haematobium* prevalence < 2% in the community-based survey and an apparent *S. haematobium* prevalence < 3% in the school-based survey, based on single urine filtration microscopy. Otherwise, the shehia will be considered a “hotspot” IU (Fig. 3). Results gained over the study period will show whether these thresholds and respective intervention packages successfully sustain and accelerate the gains and prevent recrudescence of *S. haematobium* transmission in low-prevalence IUs. The thresholds will be evaluated annually in light of the results of the cross-sectional surveys and adapted if needed.

#### Surveillance-response activities in low-prevalence implementation units

In a surveillance-response system, disease-related data are continuously and systematic collected and analysed. With constant monitoring, resurgence of transmission can be detected immediately and interventions can be implemented as a response to prevent reintroduction of the disease [4, 45].

**Fig. 4:**
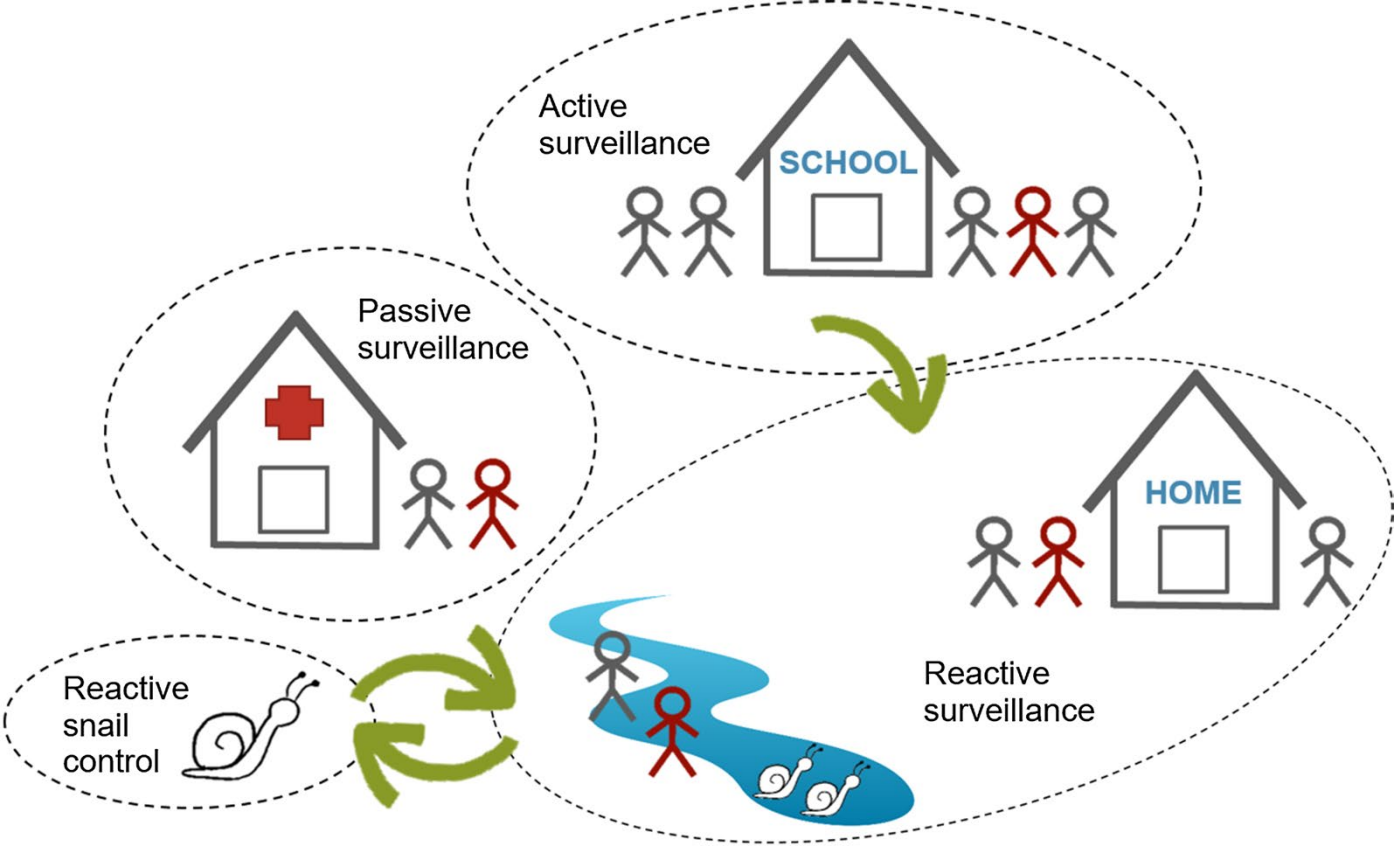
Interventions and components of surveillance-response activities implemented in low-prevalence shehias of the SchistoBreak study in Pemba, Tanzania

In our low-prevalence IUs, where large parts of the population are not infected with *S. haematobium*, the intervention approach is shifted from large-scale measures with high coverage towards targeted micro-interventions, i.e. from MDA in communities and schools to risk-based surveillance-response, including active, passive and reactive case finding, treatment of haematuria-positive and/or *S. haematobium*-infected individuals, and reactive snail control (Fig. 4).

As starting point for risk-based surveillance activities, we will select the largest public primary school in each shehia, and a madrassa with a minimum of 50 children that is located at maximum 500 m away from a water body and the farthest away from the primary school to gain a differentiated picture about the *S. haematobium* micro-epidemiology at school level in each shehia [12].

For active case finding, we will visit the selected schools at least once per year, register all children of the grades 3–5 present at the first day of the visit, and provide them with an ICF for their parents to sign. Additionally, every child aged 12 years or older will be invited to sign an assent form on their own behalf. The next day, upon submission of the signed ICF and assent forms, each child will receive a plastic container and be invited to produce its own urine sample. The submitted urine samples will be examined at the point-of-care (PoC) in the school using reagent strips (Haemastix; *Siemens* Healthcare Diagnostics AG; Zürich, Switzerland) and additional PoC tests if available. Haematuria-positive and/or *S. haematobium* infected children will be treated with praziquantel (40 mg/kg using a dose pole) on the day of or day after examination.

For reactive case detection in the days following the active case detection in schools, children tested positive in the schools will be asked to show a member of the study team their house of residence and the water bodies they are using for household chores or leisure activities. Household members of the infected child who are present at the time of the visit will be offered PoC testing with reagent strips (and additional PoC tests if available) and praziquantel treatment if diagnosed positive.

Moreover, the study team will visit the HWCSs indicated by the positive children, where infected individuals might have acquired the *S. haematobium* infection or introduced transmission, and implement mobile test-and-treat stalls where testing-and-treatment with reagent strips (and additional PoC tests if available) and praziquantel, respectively, will be offered to all eligible individuals using these water bodies. In addition to praziquantel treatment (40 mg/kg using a dose pole) of positive individuals at the HWCSs, response measures will include a thorough survey for the presence of intermediate host snails and mollusciciding of the water bodies with niclosamide as described below.

All participants included in active and reactive surveillance will have at least the following data recorded: Sex, age, travel history, time of last praziquantel treatment, and water contact behaviour.

For passive case detection, individuals presenting with signs and symptoms of urogenital schistosomiasis in the health facilities located in the study shehias, will be tested for haematuria as a proxy for *S. haematobium* infection using reagent strips and treated with praziquantel (40 mg/kg using a dose pole) if positive. The study team and staff of the NTD programme of the MoHSWEGC will train staff of the health facilities about schistosomiasis transmission, prevention, diagnosis and treatment. Staff of the health facilities will record haematuria-positive individuals, their names and location of residency, and report them at least monthly to staff of the study team.

#### Intervention packages and data collection in hotspot implementation units

In the hotspot IUs, where a considerable part of the population is still infected with *S. haematobium*, we will implement a multi-disciplinary intervention package including MDA, snail control and behaviour change communication activities.

Large-scale MDA with praziquantel is the corner stone for the control and prevention of morbidity due to schistosomiasis [3, 5]. Praziquantel treatment is administered without prior diagnosis to large parts of the population living in endemic areas. In our hotspot IUs, MDA will be conducted at least annually in communities and schools by the Zanzibar NTD Programme. In communities, community drug distributors trained by the MoHSWEGC will visit all households in a door-to-door approach and offer to treat children aged 4 years or older with praziquantel (40 mg/kg) if they do not receive treatment in schools. Moreover, they will offer praziquantel to all adults that are eligible for treatment according to national treatment guidelines. In schools, praziquantel (40 mg/kg) will be provided to all children present at the day of treatment by teachers and staff of the Zanzibar NTD Programme using a dose pole [50]. The intake of tablets will be directly observed. Treatment coverage will be reported by the Zanzibar MoHSWEGC based on reports from their staff, community drug distributors, and teachers. Moreover, we will conduct our own post-treatment coverage surveys in line with the annual cross-sectional parasitology surveys in the hotspot communities and schools. When invited to submit a urine sample for annual monitoring, individuals enrolled in the survey will also be invited to respond to an ODK questionnaire, asking if they have received and swallowed the drugs provided in the MDA treatment round preceding the survey.

Snail control with the molluscicide niclsoamide is suggested by WHO as supplementary intervention to MDA in schistosomiasis-affected areas [5, 51–53]. At each identified HWCS in the hotspot IUs, a trained team will conduct regular snail surveys. While wearing wellington boots, waiders and rubber gloves to protect them from water that potentially contains infective *S. haematobium* cercariae, the team will search for snails of all species for 10 min in 20 m of the shoreline [41]. The number of snails from each collected species and data of locality, characteristics and type of water body, and observed behavioural activities will be recorded in ODK. All *Bulinus* will be taken to the laboratory for examination for *S. haematobium* infection. At all HWCSs in water bodies where *Bulinus* spp. is found, the team will apply the molluscicide niclosamide (in a concentration of 8–10 g/litre) to clear the HWCSs from infected snails and to prevent reinfection in the human population that is treated with praziquantel against schistosomiasis. Niclosamide will be sprayed to the shorelines using backpack sprayers or a petrol sprayer and the team will wear protective gear and clothing to avoid potential inhalation and eye and skin irritation [17, 54]. The amount of niclosamide used and the time and place of application will be recorded in ODK. To reduce the impact of niclosamide on other aquatic organisms, we will only focally apply the molluscicide in areas known for human water contact behaviour but not across the whole water bodies.

Behaviour change communication and community engagement is key for the success and sustainability of interventions for schistosomiasis elimination [11]. To achieve a change in behaviour, health communication, which takes local knowledge, attitudes and practices into account and integrates the communities in priority settings for decision making and planning of schistosomiasis interventions, is essential [55]. In the hotspot IUs, we will implement behavioural interventions, which were created in a human centred design approach and successfully applied in previous studies in Zanzibar [48, 56–58]. The following intervention components will be implemented: i) installation of washing platforms at a place identified together with the community in close proximity to a clean water source (i.e. pump, tap or well) to provide access and safe alternatives to washing clothes at natural water bodies; ii) training of school and madrassa teachers in classroom-based participatory teaching methods to educate children about schistosomiasis transmission and prevention using flip-charts, snail boards, blood fluke pictures, life cycle drawing, and alternative safe play methods; and iii) school-based health communication days for schistosomiasis prevention, introducing safe play methods with health education components in schools in collaboration with health teachers and classes. Within the annual cross-sectional community- and school-based surveys, we will collect KAP data including information about the perception of schistosomiasis, use of washing platforms and alternative play options, and a potential change in behaviour to assess the impact of the behaviour change and communication measures.

### Laboratory procedures and data collection

The urine samples that are collected in the study will be examined with the following techniques for *S. haematobium* infections at the PoC, or in the laboratories of PHL-IdC, located in Chake Chake, Pemba.

In the annual cross-sectional parasitological surveys and for active, reactive and passive surveillance, urine samples will be screened for microhaematuria using reagent strips (Haemastix; *Siemens* Healthcare Diagnostics AG; Zürich, Switzerland). The colorimetric test results will be recorded semi-quantitatively (0 = negative, 1 =  + , 2 =  +  + , 3 =  +  +  + , 4 = trace).

Additionally, in the cross-sectional parasitological surveys and from a subset of samples collected during active and reactive surveillance, urine samples will be examined for the presence and number of *S. haematobium* eggs using the urine filtration method. For this purpose, urine samples will be shaken vigorously and 10 ml of each sample will be filtered through a filter-holder containing a 13 mm polycarbonate filter (Sefar, Bury, United Kingdom), using a plastic syringe [59]. All *S. haematobium* eggs present on the filter will be counted under a microscope by experienced laboratory technicians and exact egg counts will be recorded for each participant.

For external quality control, all microscope slides with the filter containing potential *S. haematobium* eggs collected during the annual cross-sectional surveys will be covered with cellophane soaked in glycerol and stored in slide storing boxes at PHL-IdC in Pemba until the end of the survey period. After each survey period, 10% of the slides will be selected based on the original electronic results by a Swiss TPH epidemiologist, sorted out locally and re-read by an external senior laboratory technician who is blinded to the results. The number of *S. haematobium* eggs counted in quality control will be recorded and compared with the original results as part of the statistical analysis done at Swiss TPH. In the case of significant discrepancies (false negatives, false positives, egg counts resulting in a different infection intensity category) in more than 20% of the re-read slides, all stored slides of the last survey will be re-read.

Both, reagent strip and urine filtration methods are not very sensitively detecting light intensity infections, as they might occur frequently in people living in elimination settings such as Pemba [29]. Hence, 10 ml from each urine samples collected at the baseline and endline survey will be stored at -20 °C and examined with a highly sensitive and specific test at the end of the study. This might be a DNA-based PCR approach [31] or an antigen-based test such as the up-converting phosphor-lateral flow circulating anodic antigen (UCP-LF) CAA assay [30] or any other test with excellent parameters developed until 2024.

To increase the performance of our surveillance-response approach, ideally a PoC test with a higher sensitivity and specificity than the reagent strips will be used. No such tests are yet (in 2021) commercially available, but several are under development. In the SchistoBreak project, we will investigate the performance of new rapid diagnostic tests, such as the Recombinase Polymerase Amplification (RPA) assay [44, 60], or other DNA-based, antigen-based or egg-based diagnostic tests for *S. haematobium* diagnosis at the PoC during our cross-sectional and surveillance activities. Once validated, applicable at the PoC, and available and affordable in sufficient numbers to screen our participants, promising candidates might be applied throughout the cross-sectional surveys and surveillance activities of the SchistoBreak project.

### Participant timeline

**Fig. 5:**
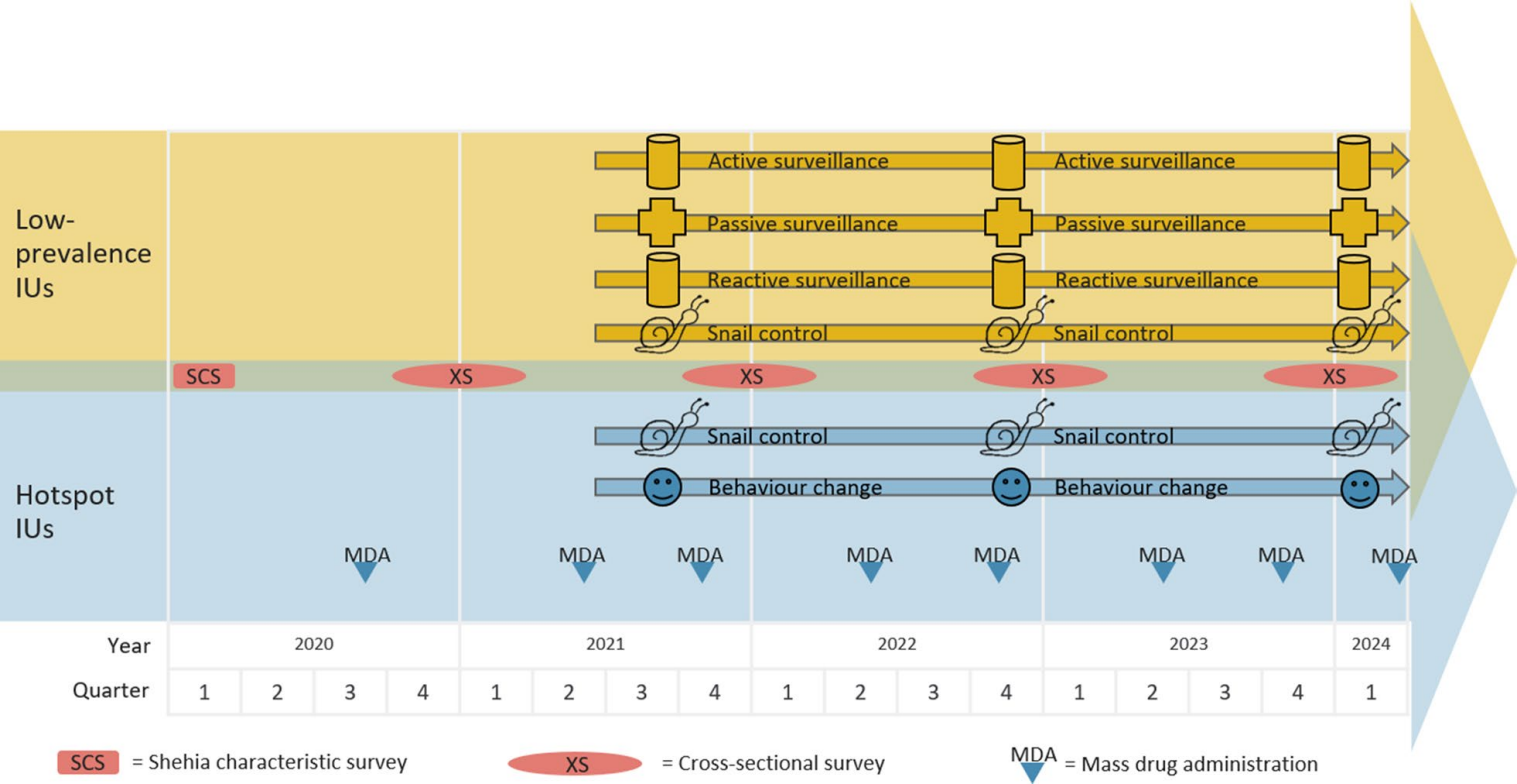
Timeline of interventions against urogenital schistosomiasis in the 20 SchistoBreak study shehias in Pemba, Tanzania. IU: implementation unit

The study will be implemented from March 2020 to June 2024, allowing for three years of interventions. Participants will be met on one or two subsequent days, for consenting, questionnaires and urine collection and examination. Figure 5 shows the timeline of surveillance-response activities in low-prevalence IUs and of the multi-pronged interventions in hotspot IUs.

### Sample size

We run a series of simulations to assess how precisely the sensitivity of the surveillance system can be estimated. We modelled different scenarios with varying prevalences in schoolchildren as well as in the general population, different values for clustering and different assumptions for diagnostic test accuracy and surveillance system performance. The results revealed that sampling an average of 140 schoolchildren and 250 community members in each of 15 low-prevalence shehias in the cross-sectional surveys enables us to estimate the prevalence with a median precision (defined as one half length of the confidence interval) of 10 to 15 percentage-points in schoolchildren and of 5 to 7 percentage-points in the general population. The bias (defined as difference between the sensitivity calculated using the apparent prevalence observed during cross-sectional surveys and the sensitivity calculated using the "true" apparent prevalence observed during the surveillance activities) had a median of zero and an interquartile range from − 0.05 to 0.05.

Hence, conducting the cross-sectional surveys in the schools and communities of all 20 study shehias, the yearly total sample is 7800 individuals (ca. 10% of the population, based on an average of 4000 inhabitants per shehia).

In addition, during the surveillance activities in 15 low-prevalence shehias, we roughly estimate to test annually about 12,150 individuals (ca. 20% of the population). Among them, around 7500 will be subjected to active surveillance in high-risk schools (estimated n = 15 shehias*1 school*450 children + estimated n = 15 shehias*1 school*50 children = 7500 samples per year), 750 to reactive surveillance in households (estimated n = 15 shehias*10 households*5 inhabitants = 750 samples per year) and 1500 at mobile test-and-treat stalls (estimated n = 15 shehias*10 water bodies *10 people = 1500 per year), and 2400 to passive surveillance in health facilities (estimated n = 20 health facilities*12 months*10 patients = 2400 samples per year).

### Data management

All data collected from individuals will include a specific personal identifier code for the individual. Laboratory data will be paper-captured before they are double entered into electronic databases by trained local data entry clerks at PHL-IdC. Double entered data will be cleaned by an epidemiologist at Swiss TPH. All discrepant electronic data will be returned to the local data entry clerks, who will trace back the personal identifier code in the original paper forms, re-enter the discrepant results and return the corrected data so that the final datasets can be generated. Registration, questionnaire and additional diagnostic data will be entered directly into the questionnaire software ODK using a tablet computer (Samsung Galaxy Tab A 2019) and subsequently uploaded on a secure server. The personal identifier code will be used to merge registration, questionnaire and laboratory examination data from each individual. Only coded data will be statistically analysed. To report the laboratory results back to the individuals, coded data can be linked with the participant names.

Data entered into electronic databases will only be accessible to authorized personnel directly involved with the study by use of a protected password. Hard copies of the source data such as student registries, laboratory record sheets, ICFs and assent forms will remain at the PHL-IdC in Pemba, respectively, for at least 10 years and stored at a safe place. The electronic versions of the data entries as well as electronic-captured data will be uploaded to and stored on a secure server from Swiss TPH.

### Statistical methods

To analyse the primary outcome, we define the sensitivity of the surveillance system as the proportion of cases detected:

$${\text{SE}} = {\text{N}}_{{{\text{inf detected by surveillance}}}} / {\text{N}}_{{{\text{inf}}}}$$

The total number of infected persons is unknown but will be estimated from the cross-sectional surveys as:

$${\text{N}}_{{{\text{inf}}}} = {\text{ P}}_{{{\text{school}}}} *{\text{ N}}_{{{\text{school}}}} + {\text{ P}}_{{{\text{non}} - {\text{school}}}} *{\text{ N}}_{{ {\text{non}} - {\text{school}}}}$$

whereby prevalences (P) and corresponding confidence intervals are estimated via generalized estimating equations for binary data with independent correlation structure to account for correlation within clusters.

The prevalence and intensity of *S. haematobium* infections will be calculated by different diagnostic approaches used in the annual cross-sectional surveys. Intensity of infection will be classified into light (< 50 eggs/10 ml urine) and heavy (≥ 50 eggs/10 ml urine) according to thresholds provided by the WHO for urine filtration results [3].

The timeliness of case notification and reactive intervention will be assessed by recording the time needed from diagnosis to treatment, from diagnosis to follow-up of *S. haematobium* positive individuals and test-and-treat of their household members, and from diagnosis to reactive snail control.

The acceptability of the surveillance-response approach will be assessed by a mixed methods approach based on the results of questionnaires run in parallel to test-and-treat approaches in low-prevalence shehias.

The coverage of test-and-treat activities will be calculated by assessing the number of people who were targeted by test-and-treat activities in relation to the total population.

MDA coverage will be calculated by assessing the number of people reporting in the cross-sectional surveys that they received praziquantel during the last MDA in relation to the total number of participants in the cross-sectional surveys.

MDA compliance in hotspot areas will be calculated by assessing the number of people reporting in the cross-sectional surveys that they have taken praziquantel as recommended, in relation to the total number of participants in the cross-sectional surveys.

The impact of snail control will be determined by assessing the snail abundance and number of intermediate host snails at each HWCS identified over time during regular snail surveys.

The impact of behaviour change interventions in schools will be evaluated by assessing knowledge, attitude and practices of schoolchildren over time based on questionnaires.

The impact of the intervention approaches on the *S. haematobium* prevalence will be analysed descriptively.

The diagnostic accuracy of new PoC tests will be calculated by determining the proportion of individuals that have been correctly identified as *S. haematobium*-infected (sensitivity) and the proportion of individuals that have been correctly identified as negative (specificity) in comparison with the reference test.

The number and location of schools, madrassas, health facilities, and water bodies and the abundance of intermediate host snails of the genus *Bulinus* identified by micro-mapping of the IUs, will be analysed descriptively.

Statistical analyses will be carried out with the statistical software STATA and R.

## Discussion

In the new WHO Roadmap on NTDs published in 2021, the global elimination of schistosomiasis as a public health problem and the validated absence of infections in humans in 25 among 78 endemic countries is set as target for 2030 [5]. To achieve these goals, countries and their schistosomiasis programme managers will need clear guidance on which intervention strategies to apply, which population groups to target, which diagnostics to use, and at what thresholds to change and adapt their strategies [11–13]. In Zanzibar, which is committed to eliminate urogenital schistosomiasis in the next years, a long-term study conducted from 2012 to 2020, revealed considerable temporal and spatial heterogeneity of *S. haematobium* infections that will need to be considered in future intervention planning [6, 17, 18]. Within the SchistoBreak study we will address the focality and heterogeneity of *S. haematobium* transmission in Pemba and aim to investigate novel adaptive intervention strategies as well as standard and new diagnostic tools for schistosomiasis elimination in Zanzibar.

Working in an elimination setting, however, there are foreseeable challenges for analyses and implementation:

First, most of the population in our study area is free of schistosomiasis and there will only be very few *S. haematobium* infected individuals. Hence, even with a very large sample size involving several thousand participants, the study is underpowered and we will most likely not be able to determine the impact of our interventions in terms of statistically significant differences in the *S. haematobium* prevalence assessed in annual cross-sectional surveys. Therefore, as a computable and meaningful primary outcome, we will assess the sensitivity of the surveillance approach, based on the number of *S. haematobium* cases detected by active, reactive and passive surveillance in schools and communities divided by the total number of infected persons estimated from the cross-sectional surveys.

Second, the urine filtration and reagent strip methods that we will apply for the standard diagnosis of *S. haematobium* infection markers are not very sensitively and specifically detecting light intensity infections as primarily found in Zanzibar [29–31, 61]. Hence, in cross-sectional surveys, as well as in the surveillance-response approach, we will likely miss a considerable number of *S. haematobium-*positive individuals and underestimate the “true” prevalence. Clearly, our surveillance system will only be as good as the diagnostic methods employed. The undetected false-negative cases missed due to low test sensitivity might act as a reservoir for infections and contribute to continuous transmission. The false-positive individuals indicated by low test specificity will be chased for nothing and valuable resources and time will be wasted [13]. To improve the surveillance and get a more realistic picture of the “true” prevalence in cross-sectional surveys, in addition to the standard methods, we will apply DNA-based or antigen-based diagnostic tests, at least for the examination of urine samples at baseline and endline and, once readily available and validated, at the PoC during surveillance.

Third, to control the morbidity due to schistosomiasis, WHO provides several thresholds based on the baseline prevalence among school-aged children to decide upon the treatment strategy and frequency of MDA [3, 4]. However, to our knowledge, with regard to the schistosomiasis elimination goals, official thresholds indicating when to increase the frequency of MDA, or stop large-scale MDA and change tactics to surveillance strategies for detecting elimination or resurgence of transmission are not yet published, but urgently needed. These intervention thresholds for elimination settings should ideally be available for or transferable to a set of different diagnostic methods, which, depending on their sensitivity and specificity, will reveal different prevalences in the same population. Cross-diagnostic threshold adaptation has been evaluated for several standard tests used for the identification of both *S. haematobium* and *S. mansoni*, but only for thresholds at 10% prevalence, with samples from school-aged children, and in settings with higher intensity of infections than in Zanzibar [62, 63]. In our study, the thresholds to stratify low-prevalence IUs and hotspot IUs and the implementation of respective intervention approaches are predicated on single urine filtration results in our cross-sectional surveys and an analytical picture of the micro-epidemiology of urogenital schistosomiasis in Zanzibar established over the past 10 years. Due to age-dependent exposure rates [6, 64], we have initially selected thresholds determining hotspot IUs at ≥ 3% prevalence in individuals aged ≥ 4 years sampled in community-based surveys and at ≥ 2% *S. haematobium* prevalence in schoolchildren sampled in school-based surveys. The suggested thresholds will be subject to change and adaptation over the course of the project, i.e. in case we observe a considerable increase of prevalence in the low-prevalence IUs targeted with surveillance response.

Finally, good coverage will be key for the success of our intervention approaches. In the low-prevalence IUs, surveillance-response activities will start in schools and be extended in a snowball system to households of *S. haematobium*-infected individuals and the water bodies they use. In hotspot IUs, MDA will be conducted in communities and target all eligible household members in a shehia. Schools, including not only primary schools, but also nurseries, secondary schools and madrassas, will be used as additional venues for MDA to reach as many children as possible. Snail surveys will be conducted repeatedly and multiple times per year at the HWCSs of all known water bodies in hotspot shehias. If *Bulinus* is detected in a water body, the HWCSs will be sprayed with niclosamide. Behaviour change communication including trainings in interactive teaching methods about schistosomiasis for teachers and their equipment with schistosomiasis teaching material will target all public, private and religious schools in the hotspot shehias. Public outreach days (Kichocho days) will be conducted in at least two schools per shehia and reach not only the majority of teachers and children attending the school but additional visitors from the communities. Striking health education messages and knowledge about the transmission and prevention of schistosomiasis will be transferred. Two laundry platforms per shehia will be constructed in close collaboration with the community near clean water sources to provide the communities with safe options for washing clothes. However, the implementation and coverage of the surveillance-response activities in low-prevalence IUs and the intervention package in hotspot IUs will be limited by time, costs and logistical feasibility and require careful and adaptive planning and implementation throughout the study period.

Despite the highlighted challenges, the SchistoBreak study will fill a critical health knowledge gap and produce important and much needed results. The study will show whether the performance of surveillance-response as intervention in low-prevalence IUs is sufficiently high to detect successfully all urogenital schistosomiasis cases and to respond with adequate interventions so that recrudescence of transmission can be prevented. It will also reveal if the *S. haematobium* prevalence in hotspot IUs can be reduced to very low levels when a comprehensive, multi-disciplinary intervention package is applied. Finally, the study will show whether new diagnostic tests are suitable for application at the PoC and outperforming current standard tests in their sensitivity and specificity to detect *S. haematobium* infections. Hence, our study will shed light on the field applicability and performance of novel adaptive intervention strategies and new diagnostic tools for schistosomiasis elimination and reveal whether progress towards interruption of transmission can indeed be achieved and sustained in the suggested way. The evidence and experiences generated by micro-mapping of *S. haematobium* infections at sub-district community level, micro-targeting of different intervention approaches, and application of novel diagnostic tools can guide future strategic plans for schistosomiasis elimination in Zanzibar and inform other countries aiming for interruption of transmission.

## Data Availability

Not applicable.

## Abbreviations

GIS: Geographical Information System
HWCS: Human water contact site
ICF: Informed consent form
IU: Intervention unit
KAP: Knowledge, attitude and practice
MDA: Mass drug administration
MoHSWEGC: Ministry of Health, Social Welfare, Elderly, Gender and Children
NTD: Neglected tropical disease
PHL-IdC: Public Health Laboratory-Ivo de Carneri
PoC: Point-of-care
RPA: Recombinase Polymerase Amplification
Swiss TPH: Swiss Tropical and Public Health Institute
UCP-LF CAA: Up-converting phosphor-lateral flow circulating anodic antigen
WASH: Water, Sanitation and Hygiene
WHO: World Health Organization

## References

[1] Colley DG, Bustinduy AL, Secor WE, King CH (2014). Human schistosomiasis. Lancet.

[2] GBD 2017 DALYs and HALE Collaborators. Global, regional, and national disability-adjusted life-years (DALYs) for 359 diseases and injuries and healthy life expectancy (HALE) for 195 countries and territories, 1990–2017: A systematic analysis for the Global Burden of Disease Study 2017. Lancet. 2018;392(10159):1859–922. (**Epub 2018/11/13**). 10.1016/S0140-6736(18)32335-3 PMC6252083 30415748

[3] WHO (2006). Preventive chemotherapy in human helminthiasis: Coordinated use of anthelminthic drugs in control interventions: a manual for health professionals and programme managers.

[4] WHO. Schistosomiasis progress report 2001–2011 and strategic plan 2012–2020. World Health Organization, 2013.

[5] WHO (2020). Ending the neglect to attain the Sustainable Development Goals—a road map for neglected tropical diseases 2021–2030.

[6] Trippler L, Ame SM, Hattendorf J, Juma S, Abubakar S, Ali SM (2021). Impact of seven years of mass drug administration and recrudescence of *Schistosoma haematobium* infections after one year of treatment gap in Zanzibar: Repeated cross-sectional studies. PLoS Negl Trop Dis..

[7] Ramzy RMR, Rabiee A, Abd Elaziz KM, Campbell CH, Kittur N, Colley DG (2020). Test, treat, track, test, and treat active surveillance toward elimination of schistosomiasis: a feasibility study. Am J Trop Med Hyg.

[8] Shortt JA, Timm LE, Hales NR, Nikolakis ZL, Schield DR, Perry BW (2021). Population genomic analyses of schistosome parasites highlight critical challenges facing endgame elimination efforts. Sci Rep..

[9] Zhou Y, Chen Y, Jiang Q. History of human schistosomiasis (bilharziasis) in China: From discovery to elimination. Acta Parasitol. 2021. (**Epub 2021/03/14**). 10.1007/s11686-021-00357-9 33713275

[10] Rollinson D, Knopp S, Levitz S, Stothard JR, Tchuem Tchuenté LA, Garba A (2013). Time to set the agenda for schistosomiasis elimination. Acta Trop.

[11] Monnier N, Barth-Jaeggi T, Knopp S, Steinmann P (2020). Core components, concepts and strategies for parasitic and vector-borne disease elimination with a focus on schistosomiasis: a landscape analysis. PLoS Negl Trop Dis..

[12] Tchuem-Tchuente LA, Rollinson D, Stothard JR, Molyneux D (2017). Moving from control to elimination of schistosomiasis in sub-Saharan Africa: time to change and adapt strategies. Infect Dis Poverty..

[13] Gass K (2020). Time for a diagnostic sea-change: Rethinking neglected tropical disease diagnostics to achieve elimination. PLoS Negl Trop Dis..

[14] WHO. Resolution on schistosomiasis WHA65.21 Geneva: World Health Organization; 2012. https://www.who.int/neglected_diseases/Schistosomiasis_wha65/en/.

[15] Toor J, Coffeng LE, Hamley JID, Fronterre C, Prada JM, Castaño MS (2020). When, who, and how to sample: Designing practical surveillance for 7 neglected tropical diseases as we approach elimination. J Infect Dis.

[16] Knopp S, Person B, Ame SM, Mohammed KA, Ali SM, Khamis IS (2013). Elimination of schistosomiasis transmission in Zanzibar: Baseline findings before the onset of a randomized intervention trial. PLoS Negl Trop Dis..

[17] Knopp S, Person B, Ame SM, Ali SM, Hattendorf J, Juma S (2019). Evaluation of integrated interventions layered on mass drug administration for urogenital schistosomiasis elimination: a cluster-randomised trial. Lancet Glob Health.

[18] Knopp S, Ame SM, Person B, Hattendorf J, Rabone M, Juma S (2019). A 5-Year intervention study on elimination of urogenital schistosomiasis in Zanzibar: parasitological results of annual cross-sectional surveys. PLoS Negl Trop Dis..

[19] Knopp S, Mohammed KA, Ali SM, Khamis IS, Ame SM, Albonico M (2012). Study and implementation of urogenital schistosomiasis elimination in Zanzibar (Unguja and Pemba islands) using an integrated multidisciplinary approach. BMC Public Health.

[20] Stothard JR, Mgeni AF, Khamis S, Seto E, Ramsan M, Rollinson D (2002). Urinary schistosomiasis in schoolchildren on Zanzibar Island (Unguja), Tanzania: a parasitological survey supplemented with questionnaires. Trans R Soc Trop Med Hyg.

[21] WHO. Atlas of the global distribution of schistosomiasis: 29 - United Republic of Tanzania. 1987.

[22] Mott KE (1989). Contrasts in the control of schistosomiasis. Mem Inst Oswaldo Cruz.

[23] Savioli L, Dixon H, Kisumku UM, Mott KE (1989). Control of morbidity due to *Schistosoma haematobium* on Pemba island: programme organization and management. Trop Med Parasitol.

[24] Savioli L, Dixon H, Kisumku UM, Mott KE (1989). Control of morbidity due to *Schistosoma haematobium* on Pemba island; selective population chemotherapy of schoolchildren with haematuria to identify high-risk localities. Trans R Soc Trop Med Hyg.

[25] Albonico M, Chwaya HM, Montresor A, Stolfzfus RJ, Tielsch JM, Alawi KS (1997). Parasitic infections in Pemba Island school children. East Afr Med J.

[26] Mgeni AF, Kisumku UM, McCullough FS, Dixon H, Yoon SS, Mott KE (1990). Metrifonate in the control of urinary schistosomiasis in Zanzibar. Bull World Health Organ.

[27] Stothard JR, Rollinson D (1997). Molecular characterization of *Bulinus globosus* and *B. nasutus* on Zanzibar, and an investigation of their roles in the epidemiology of *Schistosoma haematobium*. Trans R Soc Trop Med Hyg..

[28] Rollinson D, Stothard JR, Southgate VR (2001). Interactions between intermediate snail hosts of the genus *Bulinus* and schistosomes of the *Schistosoma haematobium* group. Parasitology.

[29] Knopp S, Ame SM, Hattendorf J, Ali SM, Khamis IS, Bakar F (2018). Urogenital schistosomiasis elimination in Zanzibar: accuracy of urine filtration and haematuria reagent strips for diagnosing light intensity *Schistosoma haematobium* infections. Parasit Vectors..

[30] Knopp S, Corstjens PL, Koukounari A, Cercamondi CI, Ame SM, Ali SM (2015). Sensitivity and specificity of a urine circulating anodic antigen test for the diagnosis of *Schistosoma haematobium* in low endemic settings. PLoS Negl Trop Dis..

[31] Keller D, Rothen J, Dangy JP, Saner C, Daubenberger C, Allan F (2020). Performance of a real-time PCR approach for diagnosing *Schistosoma haematobium* infections of different intensity in urine samples from Zanzibar. Infect Dis Poverty..

[32] Colley DG, Fleming FM, Matendechero SH, Knopp S, Rollinson D, Utzinger J, et al. Contributions of the Schistosomiasis Consortium for Operational Research and Evaluation (SCORE) to schistosomiasis control and elimination: Key findings and messages for future goals, thresholds, and operational research. Am J Trop Med Hyg. 2020;103(1_Suppl):125–34. Epub 2020/05/14. 10.4269/ajtmh.19-0787 PMC7351304 32400345

[33] Gardner JMF, Mansour NR, Bell AS, Helmby H, Bickle Q (2021). The discovery of a novel series of compounds with single-dose efficacy against juvenile and adult *Schistosoma* species. PLoS Negl Trop Dis..

[34] Bergquist R, Yang GJ, Knopp S, Utzinger J, Tanner M (2015). Surveillance and response: tools and approaches for the elimination stage of neglected tropical diseases. Acta Trop.

[35] Qian MB, Chen J, Bergquist R, Li ZJ, Li SZ, Xiao N (2019). Neglected tropical diseases in the People's Republic of China: Progress towards elimination. Infect Dis Poverty..

[36] Liang S, Yang C, Zhong B, Guo J, Li H, Carlton EJ (2014). Surveillance systems for neglected tropical diseases: Global lessons from China's evolving schistosomiasis reporting systems, 1949–2014. Emerg Themes Epidemiol.

[37] Bezerra DVF, Queiroz JW, Câmara VAV, Maciel BLL, Nascimento ELT, Jerônimo SMB. Factors associated with *Schistosoma mansoni* infestation in northeast Brazil: A need to revisit individual and community risk factors. Am J Trop Med Hyg. 2021. Epub 2021/02/17. 10.4269/ajtmh.19-0513 PMC8045610 33591939

[38] Sokolow SH, Wood CL, Jones IJ, Swartz SJ, Lopez M, Hsieh MH (2016). Global assessment of schistosomiasis control over the past century shows targeting the snail intermediate host works best. PLoS Negl Trop Dis..

[39] Ng'etich AKS, Voyi K, Mutero CM (2021). Assessment of surveillance core and support functions regarding neglected tropical diseases in Kenya. BMC Public Health..

[40] Rudge JW, Stothard JR, Basanez MG, Mgeni AF, Khamis IS, Khamis AN (2008). Micro-epidemiology of urinary schistosomiasis in Zanzibar: Local risk factors associated with distribution of infections among schoolchildren and relevance for control. Acta Trop.

[41] Pennance T, Person B, Muhsin MA, Khamis AN, Muhsin J, Khamis IS (2016). Urogenital schistosomiasis transmission on Unguja Island, Zanzibar: Characterisation of persistent hot-spots. Parasit Vectors..

[42] Souza AA, Ducker C, Argaw D, King JD, Solomon AW, Biamonte MA (2021). Diagnostics and the neglected tropical diseases roadmap: setting the agenda for 2030. Trans R Soc Trop Med Hyg.

[43] Mabey D, Agler E, Amuasi JH, Hernandez L, Hollingsworth TD, Hotez PJ (2021). Towards a comprehensive research and development plan to support the control, elimination and eradication of neglected tropical diseases. Trans R Soc Trop Med Hyg.

[44] Archer J, Barksby R, Pennance T, Rostron P, Bakar F, Knopp S (2020). Analytical and clinical assessment of a portable, isothermal recombinase polymerase amplification (RPA) assay for the molecular diagnosis of urogenital schistosomiasis. Molecules..

[45] WHO (2006). Communicable disease surveillance and response systems.

[46] Office of Chief Government Statistician Zanzibar. Zanzibar in figures 2019. 2020.

[47] Office of Chief Government Statistician Zanzibar. 2012 Population and housing census: Population distribution by admistrative areas. 2013.

[48] Celone M, Person B, Ali SM, Lyimo JH, Mohammed UA, Khamis AN (2016). Increasing the reach: Involving local Muslim religious teachers in a behavioral intervention to eliminate urogenital schistosomiasis in Zanzibar. Acta Trop.

[49] Office of Chief Government Statistician Zanzibar. Household budget survey 2014/2015. 2016.

[50] Montresor A, Engels D, Ramsan M, Foum A, Savioli L (2002). Field test of the 'dose pole' for praziquantel in Zanzibar. Trans R Soc Trop Med Hyg.

[51] King CH, Sutherland LJ, Bertsch D (2015). Systematic review and meta-analysis of the impact of chemical-based mollusciciding for control of *Schistosoma mansoni* and *S. haematobium* transmission. PLoS Negl Trop Dis..

[52] WHO (1992). The Role of mollusciciding in schistosomiasis control.

[53] Sturrock RF. Complementary snail control in chemotherapeutic-based control programs. In: Miller MJL, E., editor. Parasitic Diseases: Treatment & Control, 1989. p. 51–7.

[54] Allan F, Ame SM, Tian-Bi YT, Hofkin BV, Webster BL, Diakité NR, et al. Snail-related contributions from the Schistosomiasis Consortium for Operational Research and Evaluation program including xenomonitoring, focal mollusciciding, biological control, and modeling. Am J Trop Med Hyg. 2020;103(1_Suppl):66–79. Epub 2020/05/14. 10.4269/ajtmh.19-0831 PMC7351297 32400353

[55] Aagaard-Hansen J, Mwanga JR, Bruun B (2009). Social science perspectives on schistosomiasis control in Africa: past trends and future directions. Parasitology.

[56] Person B, Knopp S, Ali SM, A’kadir FM, Khamis AN, Ali JN, et al. Community co-designed schistosomiasis control interventions for school-aged children in Zanzibar. Journal of Biosocial Science. 2016;48(S1):S56-S73. Epub 2016/07/18. 10.1017/S0021932016000067 27428066

[57] Person B, Ali SM, A'Kadir FM, Ali JN, Mohammed UA, Mohammed KA (2016). Community knowledge, perceptions, and practices associated with urogenital schistosomiasis among school-aged children in Zanzibar, United Republic of Tanzania. PLoS Negl Trop Dis..

[58] IDEO.org. The field guide to human-centered design: IDEO.org; 2015. 192 p.

[59] Peters PA, Mahmoud AA, Warren KS, Ouma JH, Siongok TK (1976). Field studies of a rapid, accurate means of quantifying *Schistosoma haematobium* eggs in urine samples. Bull World Health Organ.

[60] Rostron P, Pennance T, Bakar F, Rollinson D, Knopp S, Allan F (2019). Development of a recombinase polymerase amplification (RPA) fluorescence assay for the detection of *Schistosoma haematobium*. Parasit Vectors..

[61] Krauth SJ, Greter H, Stete K, Coulibaly JT, Traoré SI, Ngandolo BN (2015). All that is blood is not schistosomiasis: Experiences with reagent strip testing for urogenital schistosomiasis with special consideration to very-low prevalence settings. Parasit Vectors.

[62] Midzi N, Bärenbold O, Manangazira P, Phiri I, Mutsaka-Makuvaza MJ, Mhlanga G (2020). Accuracy of different diagnostic techniques for *Schistosoma haematobium* to estimate treatment needs in Zimbabwe: application of a hierarchical Bayesian egg count model. PLoS Negl Trop Dis..

[63] Bärenbold O, Garba A, Colley DG, Fleming FM, Haggag AA, Ramzy RMR (2018). Translating preventive chemotherapy prevalence thresholds for *Schistosoma mansoni* from the Kato-Katz technique into the point-of-care circulating cathodic antigen diagnostic test. PLoS Negl Trop Dis..

[64] Toor J, Rollinson D, Turner HC, Gouvras A, King CH, Medley GF (2020). Achieving elimination as a public health problem for *Schistosoma mansoni* and *S. haematobium*: when is community-wide treatment required?. J Infect Dis..

